# New Fault Diagnosis Method for Rolling Bearings Based on Improved Residual Shrinkage Network Combined with Transfer Learning

**DOI:** 10.3390/s24175700

**Published:** 2024-09-01

**Authors:** Tieyang Sun, Jianxiong Gao

**Affiliations:** School of Mechanical Engineering, Xinjiang University, Urumqi 830046, China

**Keywords:** rolling bearing, convolutional neural network, transfer learning, noise resistance, fault diagnosis, soft thresholding, attention mechanism, small-sample dataset

## Abstract

The fault diagnosis of rolling bearings is faced with the problem of a lack of fault data. Currently, fault diagnosis based on traditional convolutional neural networks decreases the diagnosis rate. In this paper, the developed adaptive residual shrinkage network model is combined with transfer learning to solve the above problems. The model is trained on the Case Western Reserve dataset, and then the trained model is migrated to a small-sample dataset with a scaled-down sample size and the Jiangnan University bearing dataset to conduct the experiments. The experimental results show that the proposed method can efficiently learn from small-sample datasets, improving the accuracy of the fault diagnosis of bearings under variable loads and variable speeds. The adaptive parameter-rectified linear unit is utilized to adapt the nonlinear transformation. When rolling bearings are in operation, noise production is inevitable. In this paper, soft thresholding and an attention mechanism are added to the model, which can effectively process vibration signals with strong noise. In this paper, the real noise is simulated by adding Gaussian white noise in migration task experiments on small-sample datasets. The experimental results show that the algorithm has noise resistance.

## 1. Introduction

Domestic and foreign rolling bearing failure caused by the failure of key equipment is commonplace [1,2]. According to statistics, 30% of mechanical faults are caused by bearing damage, so it is of practical significance to diagnose bearing faults [3,4]. If the state of the rolling bearing is effectively monitored and its performance is accurately assessed, not only can the maintenance strategy be developed in advance, but the reliability of the equipment and the safety of industrial production can also be greatly improved, which is directly related to the interests of the enterprise. The efficient diagnosis of rolling bearing faults plays an important role in industrial applications.

Bearing fault diagnosis has been widely studied. To solve the problem of a lack of fault data in bearing fault diagnosis, Liu et al. [5] proposed a novel transfer learning strategy by driving a one-dimensional (1D) cycle-consistent generative adversarial network (Cycle-GAN) with numerical simulation. In this strategy, a bearing dynamic model is constructed to generate simulated vibration signals under normal and fault states. A 1D Cycle-GAN is devised as the transfer learning model for signal-to-signal translation. By using all simulated signals (normal and fault) and real normal signals, this 1D CycleGAN is trained to translate each simulated signal into a nearly real signal. Hu et al. [6] drew on the Siamese network and a convolutional autoencoder, and proposed a real-time bearing fault diagnosis model based on Siamese convolutional autoencoder (RBFDSCA). First, an Industrial Internet of Things (IIoT) platform is used to collect, store, and analyze bearing data. Second, to cope with the challenge of the small sample size of faulty data, the RBFDSCA model constructs a Siamese convolutional autoencoder. The Siamese convolutional autoencoder contains a positive feature extraction network, a negative feature extraction network, and a prediction network. Using the autoencoder as a building block, Wen et al. [7] presented a novel deep clustering network, named the clustering graph convolutional network with multiple-adversarial learning (c-GCN-MAL), for the intelligent fault diagnosis of various bearings. First, multiple representations of datasets, i.e., the datasets and their structured relatedness, are extracted by the autoencoder and graph convolutional network (GCN) to recognize different classes and their related information. Then, the data-adversarial and domain-adversarial are individually set by newly defining the loss function of the proposed deep network, so that its clustering and transfer ability for the new dataset can be enhanced. Hu et al. [8] proposed a masked one-dimensional convolutional autoencoder (MOCAE) for bearing fault diagnosis based on a digital-twin-enabled Industrial Internet of Things (IIoT). The model monitors the bearing data using a set of IIoT platforms. The digital twin technology is used to build a digital twin model of the bearing device, and the parameters of the digital twin model are trained by the fault data obtained from the IIoT platform. The trained digital twin model can then simulate whether the bearing is faulty. In this digital twin model, the MOCAE model is proposed for diagnosing faulty bearing signals. The MOCAE model first extracts the features from the time series signal of the bearing using a one-dimensional convolutional autoencoder, which can enhance the reconstruction ability of hidden features to make them more representative. Next, the MOCAE model automatically extracts the feature information contained in the time series signal data by self-training in order to reduce the dependence on the labeled data. Xiao et al. [9] proposed a joint transfer network based on adversarial learning and a loss function embedded with joint MMD to achieve simultaneous alignments of marginal and conditional distributions across domains. Zhao et al. [10] proposed a multisource domain transfer learning approach called the conditional weighting transfer Wasserstein autoencoder to deal with the challenges of cross-domain fault diagnosis. Different from the traditional distribution alignment idea of directly aligning the source and target domains, the proposed framework adopts an indirect latent alignment approach to achieve better feature alignment, that is, indirectly aligning the feature distribution of the source and target in the latent feature space with the help of Gaussian prior distribution. Furthermore, considering the variability of different source domains containing information about the target domain, an ingenious conditional weighting strategy is designed to quantify the similarity of different source domains to the target domain, and further help the proposed model to minimize the discrepancy in conditional distribution. Meng et al. [11] proposed a probabilistic Bayesian parallel deep learning framework for wind turbine bearing fault diagnosis to solve difficulties in feature extraction and low confidence in diagnostic results. Next, the weights and biases in the PDL framework are converted from deterministic values to probability distributions. In this way, an uncertainty-aware method is explored to achieve reliable machine fault diagnosis.

Because the diagnosed bearings have uncertain characteristics in the model and working conditions in actual application, this means that the real-time data distribution undergoes a considerable change [12]. This violates the basic assumptions of machine learning, that is, the training set and the test set of the model should satisfy the same distribution. This will greatly reduce the generalization ability of the existing model in different working conditions.

The machine works during most of its normal state [13,14,15]. Therefore, the mechanical failure of the data sample is more difficult to collect than the normal state of the data sample. Transfer-learning-based fault diagnosis methods are more suitable for mechanical equipment fault diagnosis in domain-different cases than other deep learning methods. A domain-different case refers to a case where the source and target domain fault data do not follow the same distribution assumption [16]. The cross-domain diagnosis approach that utilizes transfer learning enhances the model’s capacity to learn the target task by borrowing diagnostic knowledge from the relevant source domain. This allows for addressing the issue of limited training data by transferring pertinent knowledge from other datasets that contain ample supervisory information. Currently, transfer learning is widely applied to the fault classification problem [17].

In recent years, there has been more and more research on transfer learning, and its application in the field of fault diagnosis has been developed rapidly [18]. Yang et al. [19] aligned the data distribution in the domain by applying the correlation alignment algorithm, then extracted the data features by using the statistical algorithm and the wavelet scattering network, and, finally, trained a nearest-neighbor classifier to classify faults of the samples in the target domain by using the feature vector matrix of the source domain. Thus, the problem of uneven distribution of bearing data was solved. Li et al. [16] added the adversarial idea of domain adaptation on the basis of transfer learning and improved the invariant features of adversarial domain adaptive learning by attaching a correlation alignment algorithm in order to further reduce the distributional differences between the data in the source domain and in the target domain. Meanwhile, in order to avoid the antagonism of a single fixed parameter, the gradient inversion layer was introduced to realize gradient inversion, and the problem caused by sample difference under different variable conditions was finally solved. Thus, the model has a higher diagnostic accuracy and generalization ability. Lee et al. [20] proposed a multiobjective instance weighting-based transfer learning network, which integrates transfer learning into the instance weighting strategy, and minimizes the distribution difference between two domains of the transfer learning model through two metrics, which are, namely, KL (Kullback–Leibler divergence) and the MMD (maximum mean discrepancy) to minimize the distributional difference between the two domains of the migration learning model. It achieves the improvement of the accuracy of equipment fault diagnosis using data from different conditions when only small amounts of data are collected from the target system conditions. Wang et al. [21] combined transfer learning with machine learning and proposed a methodology based on factor analysis, which searches a new feature space in different domains corresponding to various operating conditions, then migrates the original features to a low-dimensional latent space through feature extraction, minimizes the domain differences through the learned features, and finally saves the parameters and uses them to construct an autonomous diagnostic model for machinery based on machine learning techniques. It solves the problem of data samples following different distributions under various operating conditions. Yang et al. [22] constructed a diagnostic model using the polynomial-kernel-induced MMD distance metric to reuse the diagnostic knowledge from one machine to another, and the proposed method was validated by two transfer learning cases, which effectively reduces the distributional differences of the collected data.

With the development of transfer learning, a number of scholars have combined neural networks with transfer learning and have achieved many results [23]. Song et al. [24] trained a fault diagnosis model by introducing a long- and short-term memory network, then fine-tuned the model parameters by using a small amount of data in the target domain, and suppressed the overfitting by using improved transfer learning of elastic networks. The proposed method effectively solves the problem of acquiring the data of bearings under the condition of variable load. Zhu et al. [25] integrated convolutional neural network and transfer learning, adjusting the two layers of task-specific features in a layer-by-layer manner to standardize parameters of convolutional neural network. Then, the loss between domains was calculated by a linear combination of multiple Gauss kernels, in order to reduce the distribution difference and learn transferable features. This eventually solves the time-consuming and expensive problem of collecting tag data and building models from scratch in real industry. Li et al. [26] proposed a deep-learning-based approach using a class of weighted-domain adversarial neural networks for local domain adaptation, and proposed a class of weighted adversarial neural networks in order to encourage positive migration of shared classes and ignore source outliers to solve the partial transfer learning problem in bearing fault diagnosis. Liu et al. [27] proposed a power equipment fault diagnosis method based on energy spectrogram and deep learning. By introducing the transfer learning strategy and adding the channel domain, an attention mechanism is added to the channel feature fusion layer, with the accuracy of detection up to 99.4%, and the amount of parameter calculation greatly reduced to one-fifth of that of VGG.

Zhang et al. [28] improved the convolutional neural network in order to solve the problem of lacking data in real industry. By reducing the number of parameters to be trained in the network, and then training and fine-tuning the model through the source and target domain samples, respectively, the model’s migration was realized, and the diagnostic performance of the model was experimentally demonstrated to be good.

The research fervor of transfer learning is gradually rising, especially in the field of fault diagnosis. Due to its inherent advantages for small-sample data, transfer learning can maintain strong diagnostic performance in the face of limited fault data in actual industrial production settings. As a result, many researchers have achieved significant advancements, both in enhancing transfer learning methods themselves and in integrating them with deep learning network models. 

In this paper, a new model with excellent performance is proposed, which combines with transfer learning to improve the accuracy of rolling bearing fault diagnosis. The model adopts an adaptive residual contraction module to extract multiscale features by using different scale information in the signal, effectively alleviating the problems of gradient disappearance and gradient explosion during training. The APReLU (Adaptive Parametric Rectified Linear Unit) activation function is introduced to perform different nonlinear transformations for different vibration signals. This not only preserves the multiscale features of the input data, but also enhances the feature extraction ability of the model. Soft thresholding and attention mechanism are used to effectively process strong noise and highly redundant vibration signals, and white Gaussian noise is added to the small-sample dataset for noise experiments to verify the noise resistance of the model. The unique feature of the algorithm is the introduction of the APReLU activation function, which opens up a new idea for the research of this knowledge field. The novelty of this paper lies in the addition of Gaussian white noise to the small-sample dataset for noise experiments to verify the noise resistance of the model. 

The remainder of this paper is organized as follows. The main theories of the proposed model are introduced in Section 2. The predictive performance of the proposed model is verified by conducting experiments on the CWRU bearing dataset in Section 3. Experiments based on the JNU bearing dataset are conducted in Section 4. Some main conclusions are drawn in Section 5.

## 2. Methods and Theory

In the subsequent text, a new fault diagnosis method for rolling bearings based on adaptive residual shrinkage network combined with transfer learning is proposed. First, the basic model framework used in this research and the used theoretical methods are introduced in Section 2.1. Subsequently, basic principle and classification of transfer learning are introduced separately in Section 2.2 and Section 2.3. Finally, fine-tune-based transfer learning is introduced in Section 2.4. 

### 2.1. The Model Framework Used in This Research and the Used Theoretical Methods

**Figure 1:**
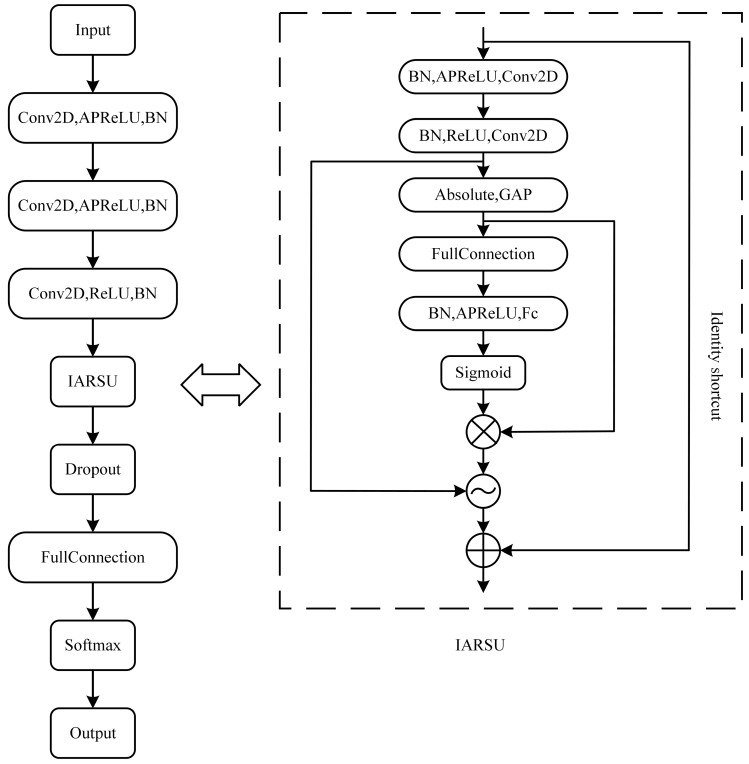
The IARSN network structure.

The model utilized in this paper is the IARSN (Improved Adaptive Residual Shrinkage Network) model, which integrates the residual shrinkage architecture with the APPeLU (Adaptive Parametric ReLU) activation function. Its main components include convolutional layers, BN (batch-normalization) layers, APReLU activation layers, ARSU (Adaptive Residual Shrinkage Unit) structure, dropout layers, fully-connected layers, global average pooling layers, and so on. The architecture of the IARSN network is illustrated in Figure 1.

In the subsequent text, the specific structures of the model are introduced. First, the residual shrinkage structure is introduced in Section 2.1.1. Subsequently, APReLU is introduced in Section 2.1.2. Finally, the convolutional layer, the global average pooling layer, and the fully-connected layer are introduced in Section 2.1.3.

#### 2.1.1. The Residual Shrinkage Structure

**Figure 2:**
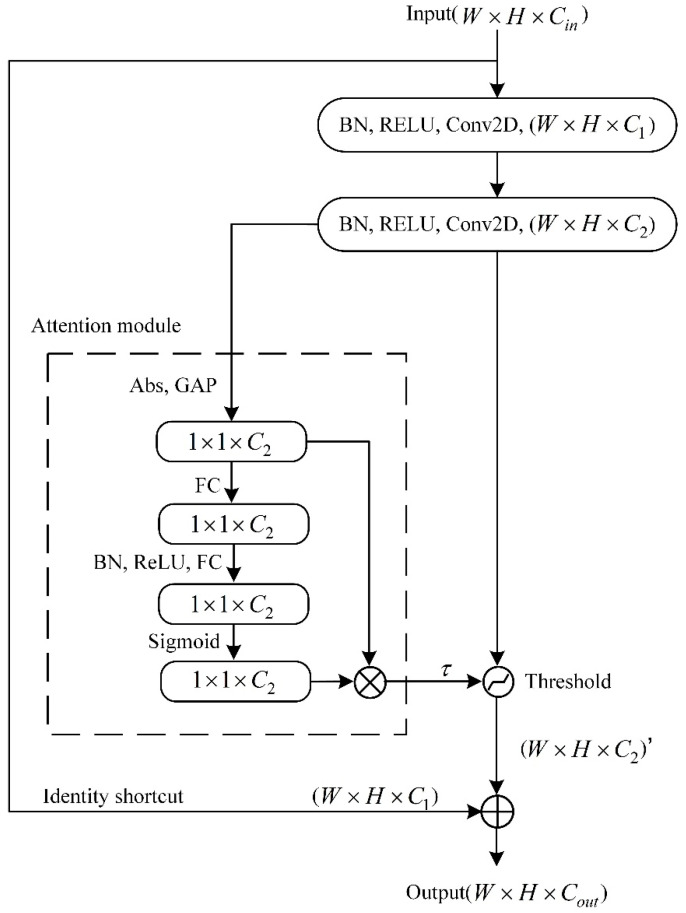
The residual shrinkage structure.

The residual contraction structure, which incorporates the attention mechanism and soft thresholding, is illustrated in Figure 2. This structure is effective for dealing with vibration signals with strong noise and high redundancy.

The attention mechanism draws on the squeeze-and-excitation network proposed by Hu et al. [29]. It uses small subnets, and a set of weights is obtained through the path of “Global pooling layer → Fully-Connected layer → ReLU function → Fully-Connected layer → Sigmoid function”. Then, this set of weights is multiplied by the characteristics of each channel to adjust the size of each channel feature.

Soft thresholding is the core step of noise reduction. The soft threshold function expression is as shown in Equation (1) [30]:

(1) y=x+τ   x<−τ  0   −τ≤x≤τx−τ   x>τ

where x is the input characteristic, y is the output characteristic, and τ is the threshold.

The soft threshold function sets the characteristic of [−τ,τ] to 0, and makes the feature that is farther away from 0 contract toward 0. Compared to ReLU, the soft threshold function does not set all negative features to zero; thereby, useful negative characteristics can be preserved. Additionally, the derivative of output over input is either 1 or 0, effectively preventing the problem of gradient disappearance and gradient explosion.

The threshold τ obtained by the subnet is a set of vectors. Its number of elements is equal to the number of feature map channels $C_{2}$ output by the second convolutional hidden layer. Multiplying each element by the corresponding channel’s feature map is performed to give different sizes of attention to each feature channel. Then, the soft threshold function is used to perform shrinkage noise reduction.

#### 2.1.2. Adaptive Parameter ReLU Activation Function

Adaptive parameters allow for more flexibility in processing different types of data. The APReLU activation function introduces nonlinearity and automatically adjusts its activation threshold. It enables the network to learn more complex mapping relationships, improves the ability of model representation, and alleviates the problems of gradient disappearance and explosion. APReLU enhances the model fitting ability and reduces the problem of dead neurons and improves training stability. 

#### 2.1.3. Introduction of Convolutional Layer, Global Average Pooling Layer, Fully-Connected Layer

Convolutional layer: Convolutional layers learn and extract features from input data. These range from low-level features to advanced features. Each convolutional layer may learn different features. The neurons in the convolutional layer are not fully connected to all the neurons in the previous layer, but are only connected to those in local regions. This local connectivity enables the convolutional layer to effectively capture local features of the input data and reduce the number of parameters in the network. All neurons use the same convolutional kernel for convolution operations. This not only reduces the number of parameters that need to be learned, but also makes the network invariable to the translation and scale change of the input data. The data input to the convolutional layer are used to extract features through convolution operation.

Global average pooling layer: This layer conducts global information extraction by pooling the spatial dimensions of the feature map into a single value. It summarizes the global information throughout the entire feature map, reduces the number of parameters, and serves as a safeguard against overfitting.

Fully-connected layer: The feature maps are flattened and mapped to the feature space. Each neuron is connected to all the outputs of the previous layer, allowing for the synthesis of all features. The fully-connected layer is able to combine higher-level feature representations into higher-level abstract features to better capture complex patterns and laws of data. It helps the model learn nonlinear relationships among features. Parameter learning enables the model to better fit the training data and have better generalization ability. The combination of the fully-connected layer with other types of layers is performed to reduce the number of parameters. The final fully-connected layer is also capable of transforming features into probability distributions for different classes, thus enabling the classification task.

### 2.2. The Basic Principle of Transfer Learning

Transfer learning generally refers to the learning process that exploits the similarities between old and new data or old and new models and transfers models that have been learned and applied in the old domain to a new domain. The relevant knowledge and theories involved in transfer learning are described as follows:

The object of transfer learning is known as the domain, which typically consists of data, data labels, and the relationships among data.

There are many forms of transfer learning, which generally involves at least two domains called the source domain and the target domain. The source domain has sufficient labeled data and is a known domain. The neural network is basically trained on the source domain. However, the target domain often lacks sufficient labels or even does not have labels. After training on the source domain data, the classifiers are applied to the target domain, and the effectiveness of the application is guaranteed by a migration algorithm.

The task of transfer learning refers to the goal of learning, and the task usually consists of labels and the function corresponding to the labels. The labeling space is known as Y, and the learning function is generally denoted as f(x).

A domain in transfer learning contains the feature space χ and the edge probability distribution P(X). A domain is denoted by D, that is, $D=\left\{\chi ,\right.\left.P(X)\right\}$. A task T contains the label space Y and the corresponding conditional distributions $P\left(Y|X\right)$. The conditional distributions are learned from the training data. 

In the process of transfer learning, the source domain $D_{s}$ and the target domain $D_{t}$, which differ from each other, are somehow connected. Based on the similarities of the two domains, a classifier that can adapt to both domains is derived to accomplish the prediction of the target task in the target domain.

### 2.3. Classification of Transfer Learning

**Figure 3:**
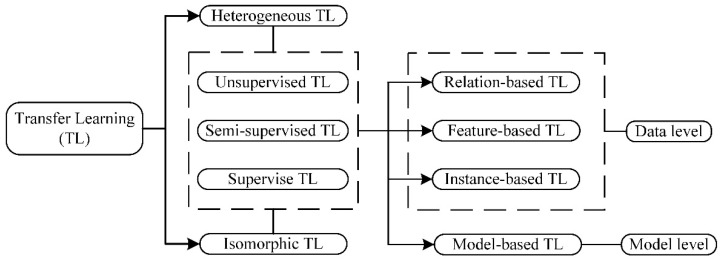
Types of transfer learning.

In recent years, a large number of scholars have invested a great deal of effort in the field of transfer learning [31], and have made some progress in many aspects. Based on this, transfer learning is divided according to different levels. It is shown in Figure 3.

As shown in Figure 3, transfer learning is classified into three main types at the data level: The first category is relation-based migration. This type is relatively not widely used. Its main idea is to conduct analogy and migration by mining the relations between domains. The second category is feature-based migration, which assumes that the features of both samples are not in the same space, or are not similar in the original same space. Thus, it can be transformed into the same feature space to make the features as similar as possible. The third category is instance-based migration. The weight is determined according to the degree of similarity of the samples. We can determine the categories of the samples based on the weight. The higher the similarity of the samples, the higher the weight. Accordingly, the migration task is completed simply and directly.

The three migration approaches mentioned above are all at the data level. There are also migration approaches at the model level. One of the most common approaches in neural networks is to perform network migration directly by sharing some parameters of the neural network. Fine-tune, which is commonly used in the field of transfer learning, is a good example of the idea of model migration.

### 2.4. Fine-Tune-Based Transfer Learning

To achieve effective supervised transfer learning, the most effective and simplest method is model-based parameter migration, and that is fine-tuning model parameters. Since the source domain data are usually relatively sufficient, the trained model based on the source domain $\theta_{S}$ has already learned a large amount of reliable knowledge from the data. By migrating this reliable knowledge to the isomorphic target domain, a more accurate target domain model can be obtained with a small amount of labeled target domain data, $\theta_{T}$. In this study, we examine the migration performance of the IARSN network by fine-tuning.

**Figure 4:**
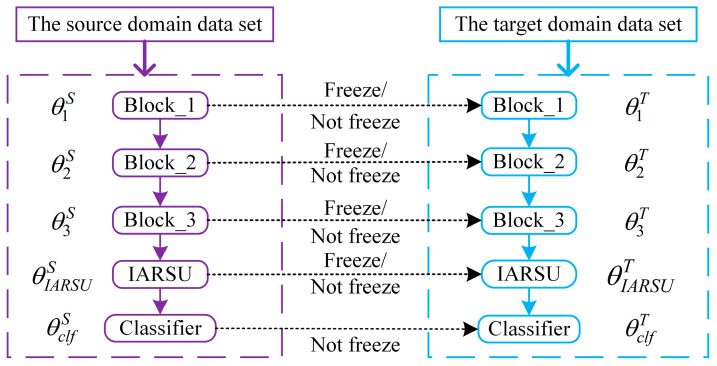
The process of fine-tuning.

With the deepening of network layers, the model will extract more complex high-level features. The network module containing a convolutional layer, a BN layer, and an activation function layer is denoted as a feature extraction module, denoted as $f_{i}(\cdot )$. The core module of the IARSN model, which is the IARSU module, is denoted as $f_{IARSU}(\cdot )$. The IARSN model contains one IARSU module and three feature extraction modules, and the feature extractor of the model can be denoted as $f(x)=f_{IARSU}(f_{3}(f_{2}(f_{1}(\cdot ))))$. The parameters of the IARSU module are assumed to be $\theta_{IARSU}$, the parameters of one single feature extraction module are assumed to be $\theta_{i}$, and the parameters of the classifier are assumed to be $\theta_{clf}$. During the fine-tuning process of model parameter migration, some modules in the front parts of the network are usually frozen on demand. In this study, the parameters of Block_1, Block_2, and Block_3 are frozen, while the parameters of the IARSU module $\theta_{IARSU}$ and the parameters of the classifier $\theta_{clf}$ are not frozen. The process of model fine-tuning is shown in Figure 4.

In Figure 4, the parameter set $\theta^{S}=\left\{\theta_{1}^{S},\theta_{2}^{S},\theta_{3}^{S},\theta_{IARSU}^{S}\right.,\left.\theta_{clf}^{S}\right\}$ denotes the network parameters of the model trained on the source domain data, while $\theta^{T}=\left\{\theta_{1}^{T},\theta_{2}^{T},\theta_{3}^{T},\theta_{IARSU}^{T}\right.,\left.\theta_{clf}^{T}\right\}$ is the initial parameters of the network in the target domain, and $\theta^{S}=\theta^{T}$ is satisfied before the model migration. The whole fine-tune process is used to train the model with a small amount of labeled target domain data in the form of partial freezing to make the target domain classification loss as small as possible. The expression of the corresponding relationship is shown in Equation (2):

(2) $$\theta^{T*}=\left\{\theta_{1}^{T}\right.,\theta_{2}^{T},\theta_{3}^{T},\theta_{IARSU}^{T},\left.\theta_{clf}^{T}\right\}=\arg\min L(y,\hat{y})$$

where $\theta^{T*}$ denotes the model parameters obtained after transfer learning; L(⋅) denotes the loss function; y denotes the target domain label value; $\hat{y}$ denotes the model predicted value.

## 3. Experiments Based on the CWRU Bearing Dataset and Results and Discussion

In the subsequent text, a set of experimental data is used to validate the proposed method. First, the CWRU bearing dataset is introduced in Section 3.1. Subsequently, the migration experiments on a small-sample target domain and migration experiments of load change are carried out in Section 3.2. Finally, the fault diagnosis experiments of rolling bearing under noise conditions are carried out in Section 3.3.

### 3.1. Presentation of Experimental Data

**Figure 5:**
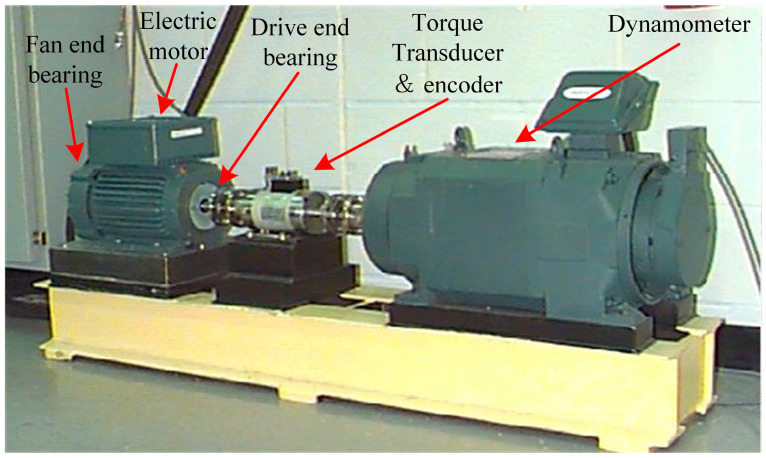
CWRU rolling bearing test bed [32].

In the experiments, the bearing data used are from the CWRU bearing dataset, which includes experimental fault data under different loads and speeds [32]. The CWRU bearing test bed is mainly equipped with devices such as a dynamometer, a torque transducer, and an electric motor. In order to obtain the bearing fault data, the EDM technology is used to make the outer-ring, the rolling-element, and the inner-ring parts produce different forms of fault. The CWRU bearing test bed is shown in Figure 5.

The CWRU bearing dataset is a publicly available dataset provided by the Case Western Reserve University bearing data center. It includes both normal and fault bearing data. To evaluate the model’s fault diagnosis performance under various working conditions, different sets of data from the CWRU public dataset are chosen for experimental comparison and analysis. The dataset is gathered from the drive-end bearing with a 6205-2RS JEM SKF model. Detailed information about the selected dataset can be found in Table 1.

In this experiment, the bearing data with 12 kHz sampling frequency are selected. According to different motor load (motor speed is determined by the motor load), the datasets under three loads are selected as the datasets of A, B, C, and the specific information is shown in Table 2.

There are a total of ten types of data selected, including nine fault data in different positions and speeds, as well as one vibration signal in the normal state. Different labels are assigned according to the different types, and detailed information is provided in Table 3. A total of 1500 samples are selected based on the working conditions corresponding to each type of motor load, with each sample containing 1024 data sampling points. The total number of samples is 4500, divided into training and test sets in a ratio of 2:1.

### 3.2. Migration Experiments on a Small-Sample Target Domain and Migration Experiments of Load Change

The network has a total of five modules. In order to obtain a better migration effect, the experiment is carried out by freezing the first three modules of the network and fine-tuning the two remaining modules, which is the basic practice of migration experiments on a small-sample target domain and migration experiments of load change. In the subsequent text, migration experiments on a small-sample target domain are firstly conducted in Section 3.2.1. Subsequently, migration experiments of load change are carried out in Section 3.2.2.

#### 3.2.1. Migration Experiments on a Small-Sample Target Domain

**Figure 6:**
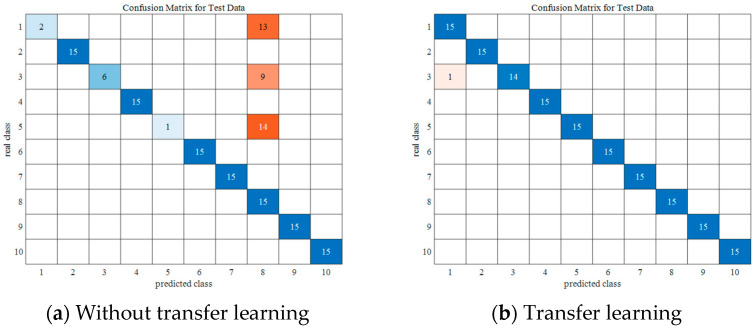
Confusion matrices.

In this experiment, three datasets, A, B, and C, are selected to perform fault diagnosis together in order to characterize the diagnostic ability of the model in the case of data complexity. In order to realize the fault diagnosis with small samples, only 10% of the data are selected for the experiments, and a total of 10 experiments are conducted. In order to not lose generality, the average of the results of 10 experiments is selected as the final result. The confusion matrices before and after migration are shown in Figure 6.

The following conclusions can be drawn from Figure 6:

The adoption of transfer learning effectively improves the performance of IARSN on the CWRU small-sample dataset. The model without transfer learning only achieves 76.3% accuracy on the test set, while the model’s accuracy is up to 99.3% on the test set with the adoption of transfer learning. This proves that the source domain dataset and the target domain dataset have common features, and this validates the migration capability of the IARSN model, which is able to learn efficiently on small-sample datasets.

#### 3.2.2. Migration Experiments of Load Change

In this section, migration experiments for different motor loads are conducted. When the dataset is used as the target domain, its sample size is selected as only 10% of the dataset, and then the trained model is migrated to this small-sample dataset. A total of six migration tasks (A–B, B–A, A–C, C–A, B–C, and C–B) are constructed for this experiment. To reduce chance effects, ten experiments are carried out for each migration task in this section and their respective accuracies are presented in Table 4.

The following can be found from Table 4:

(1) The classification accuracy of each task in ten experiments reaches more than 95%, and the fluctuation of the accuracies of each task is not big; it is between 95% and 100%. This proves the robustness of the model after migration.

(2) By comparing the fault classification accuracies of A–B, B–A; A–C, C–A; B–C, C–B, respectively, it can be found that the classification accuracies of dataset A, dataset B, and dataset C do not have a big difference when they are the source domain and the target domain of each other, from which it can be deduced that the migration of the model is not unidirectionally effective.

### 3.3. Experiments on Fault Diagnosis of Rolling Bearing in Small-Sample Dataset under Noise Conditions

The external environment and the equipment’s own structure and other factors lead to inevitable noise when rolling bearings are in operation. In order to simulate the noise pollution in the industrial environment, the Gauss white noise is always added to the diagnostic signal to test the noise resistance [33]. If the noise resistance of the proposed method is not good, it will reduce the accuracy of bearing fault diagnosis. 

The signal-to-noise ratio is defined as follows in decibels (dB) by using Equation (3):

(3) $$SNR=10\log_{10}(\frac{P_{signal}}{P_{noise}})$$

where $P_{signal}$ denotes the power of the original signal, and $P_{noise}$ denotes the power of the noise signal.

In this experiment, noise levels of −3 dB, −1 dB, 0 dB, 1 dB, and 3 dB are added to three datasets—A, B, and C. To realize small-sample fault diagnosis under noisy conditions, only 10% of the data are selected as the target domain, and the trained model is then transferred to this small-sample dataset. To ensure the generality of the findings, the average of 10 experimental results is used as the final result. The fault diagnosis accuracies for rolling bearings under different noise conditions are all above 85%, as shown in Table 5.

The results show that the IARSN model has strong antinoise capability. Under the function of adaptive activation function and residual contraction module, the model can adaptively adjust the weight information of features contained in the network, effectively extract rich hidden features, and suppress the interference of noise.

The IARSN model can set soft threshold adaptively, automatically adjust the threshold size according to the characteristics of each sample, and shrink through the soft threshold function to reduce noise, so as to better deal with different levels of noise, which makes the IARSN model have great flexibility in dealing with tasks in various noisy environments.

## 4. Experiments Based on the JNU Bearing Dataset and Results and Discussion

In the subsequent part, the proposed method is validated through experiments using experimental data from the JNU bearing dataset. Firstly, the JNU bearing dataset is introduced in Section 4.1. Subsequently, migration experiments using the JNU bearing dataset are carried out in Section 4.2. Finally, comparative experiments are carried out in Section 4.3.

### 4.1. Introduction to the JNU Bearing Dataset

**Figure 7:**
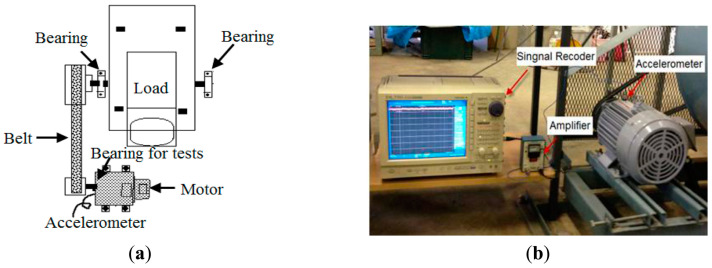
Experimental setup for the rolling bearing fault diagnosis. (**a**) Illustration of the rotation machinery and (**b**) the motor in the field [34].

For the JNU (Jiangnan University) bearing dataset, an inductor motor (Mitsubishi SB-JR) used in a centrifugal fan system is employed for the faults diagnosis test [34]. The nameplate of the machine is a 3.7 kW three-phase induction motor, with Vmax = 220 V, P = 4 pole pairs, and rated speed S = 1800 rpm. Rated slip and frequency are 6.5% and 60 Hz. The rotor is carried by two bearings, one of which is defective. Figure 7 presents an illustration and a photo of the experimental setup used for rolling bearing fault diagnosis. An accelerometer (PCB MA352A60) with a bandwidth from 5 Hz to 60 kHz and a 10 mV/g output is used to measure the vertical vibration signals in the normal, outer-race defect, inner-race defect, and roller element defect states, respectively [34]. The vibration signals measured by the accelerometer are amplified by the sensor signal conditioner (PCB ICP Model 480C02) before being transformed into the signal recorder (Scope Coder DL750) for analysis.

In the JNU bearing dataset, tests are conducted on two types of bearings: NU205 and N205 [34]. Data collection is carried out using a single accelerometer with a sampling frequency of 50 kHz over a duration of 20 seconds. The dataset comprises four categories: inner-race fault, rolling-element fault, outer-race fault, and normal condition. The inner-race fault data are obtained from the outer ring of a detachable NU205 bearing, while data for the rolling-element fault, outer-race fault, and normal condition are collected from an N205 bearing. Each fault type is recorded under three different operating conditions, corresponding to rotational speeds of 600 rpm, 800 rpm, and 1000 rpm. During the experiment, a moving window of 1024 sample points is used to capture each sample. The specific parameters of these bearings are provided in Table 6. 

### 4.2. Migration Experiments Using the JNU Bearing Dataset

In this experiment, data under the conditions of 600, 800, and 1000 rpm from the JNU dataset are utilized. Four distinct types of data are selected, comprising one normal vibration signal and three types of fault data (inner-sphere fault, rolling-element fault, and outer-sphere fault). The types of each kind of data are denoted as 1–10, respectively, as depicted in Table 7. 

**Figure 8:**
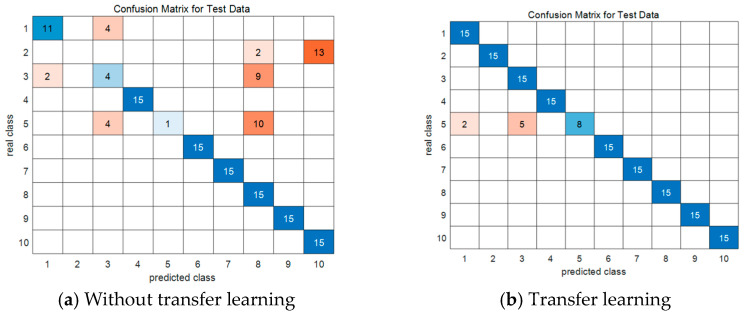
Confusion matrices.

In this experiment, the CWRU dataset is used as the source domain dataset, and the JNU dataset is used as the target domain dataset. A total of 4500 samples are selected from the CWRU dataset, and the training dataset and test dataset are divided by 2:1. To simulate the rarity of the target domain label under realistic conditions, the training dataset and the test dataset are divided by 2:1 by taking 450 samples from all kinds of label data in the JNU dataset. The IARSN model is first trained on the CWRU dataset, and then the trained model is transferred to the small-sample JNU dataset. The first three modules are frozen, and the experiment is conducted ten times. In order to not lose generality, the average value of ten experimental results is taken as the final result. The confusion matrices before and after migration are shown in Figure 8. 

As shown in Figure 8, the accuracy of the model without transfer learning on the test dataset is only 70.7%, while the model with transfer learning achieves an accuracy of 95.3% on the test dataset. This demonstrates the effectiveness of transfer learning in the IARSN model, and also proves that the model introduces APReLU to perform different nonlinear transformations on different vibration signals, retaining the features of different scales of input data. The feature extraction capability of the model is enhanced.

### 4.3. Comparative Experiments of Bearing Fault Diagnosis

**Figure 9:**
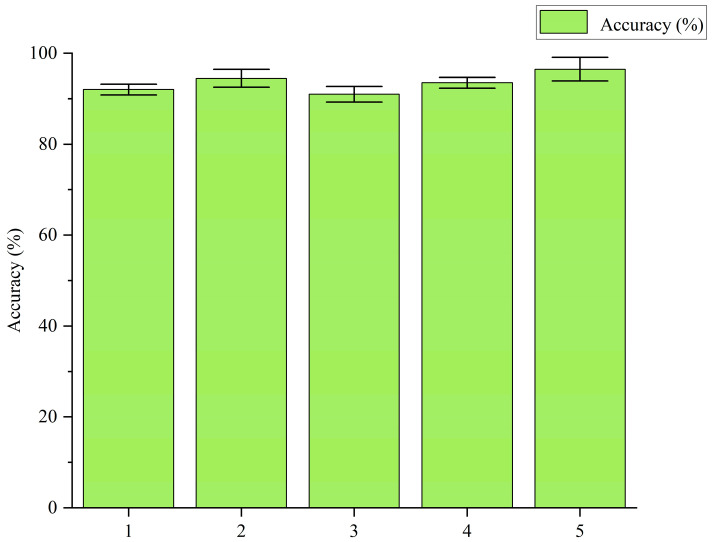
Comparative experiment results.

In order to further verify the bearing fault diagnosis performance of the proposed IARSN model, IARSN is compared with Bayesian PDL [11], GPLVM-mcODM [35], SA-ACGAN [36], and RDPN-FCDAE [37] models, respectively. The average results of ten experiments are taken as the final results. The experimental results are shown in Figure 9. In the figure, “1” refers to RDPN-FCDAE; “2” refers to SA-ACGAN; “3” refers to GPLVM-mcODM; “4” refers to Bayesian PDL; “5” refers to IARSN.

As can be seen from the figure, the IARSN model proposed in this paper has the best performance; it can adaptively adjust the weight information of the features contained in the network under the function of adaptive activation function and residual contraction module, and it can effectively extract features of vibration signals in different states. The accuracy of SA-ACGAN is higher than that of Bayesian PDL because SA-ACGAN adaptively adjusts the generator loss value by measuring the relative performance of the discriminator and generator, which makes the training convergence faster and the generated data quality better. The effect of RDPN-FCDAE is inferior to that of Bayesian PDL, probably because of the deviation in converting the original vibration signal into a time–frequency image through continuous wavelet transform (CWT). GPLVM-mcODM has the lowest accuracy because its reduction in redundancy between features is limited.

## 5. Conclusions

To address the challenge of reduced fault diagnosis accuracy due to insufficient bearing fault data, the proposed model introduces the transfer learning method. The Case Western Reserve University bearing dataset was used for training. The trained IARSN model was transferred to the scaled-down small-sample dataset and the small-sample Jiangnan University bearing dataset, the migration experiments of different loads were carried out on the small-sample target domain, and the migration experiments of different speeds were carried out on the small-sample Jiangnan University bearing dataset. The experimental results show that the proposed method improves the accuracy of rolling bearing fault diagnosis.

Soft threshold and attention mechanism were added to the algorithm, which can effectively process vibration signals with high redundancy and strong noise. The APReLU activation function was introduced to perform different nonlinear transformations for different vibration signals. This not only preserves the multiscale features of the input data, but also enhances the feature extraction capability of the model. Gaussian white noise of −3, −1, 0, 1, and 3 dB was added to the small-sample dataset to simulate real noise. The purpose was to carry out rolling bearing fault diagnosis experiment with a small-sample dataset with noise. The experimental results showed that the rolling bearing fault diagnosis accuracy using a small-sample dataset with noise is high in this model. The model has strong antinoise ability.

The uniqueness of this study lies in the integration of the model with transfer learning, the application of soft thresholding and attention mechanism, the introduction of the APReLU activation function, and the investigation of the model’s noise resistance in small-sample datasets. It opens up a new direction for the study of this field.

The future research directions of this study include the following:

(1) The used bearing data in this study are from Case Western Reserve University in America, and the experiments were based on laboratory data. It is hoped that data on bearings in actual operation will be collected for experiments in the future.

(2) This study only focuses on the ten fault states of rolling bearings to perform experiments. It is hoped that we can conduct further research on the coexistence of multiple fault states and the specific degree of failure in the future.

(3) In this paper, the vibration signal was used as the training and testing samples to study the fault diagnosis method of rolling bearings. In the future, we plan to integrate the sensing information such as temperature, current, and voltage, and to improve the robustness of the fault diagnosis method through comparison and optimization.

## Figures and Tables

**Table 1:** Specific parameters of rolling bearing.

Outer Diameter (mm)	Inner Diameter (mm)	Thickness (mm)	Rolling Body Diameter (mm)	Pitch Circle Diameter (mm)
52.00	25.00	15.00	7.94	39.04

**Table 2:** Dataset information under different operating conditions.

Dataset	Motor Load (hp)	Motor Speed (r/min)	Sampling Rate (kHz)	Fault Type
A	0	1797	12	10
B	1	1772	12	10
C	2	1750	12	10

**Table 3:** Rolling bearing fault information.

Tab	Load (HP)	Fault Type	Fault Size (Inches)
1	0, 1, 2	B07	0.007
2	0, 1, 2	B14	0.014
3	0, 1, 2	B21	0.021
4	0, 1, 2	I07	0.007
5	0, 1, 2	I14	0.014
6	0, 1, 2	I21	0.021
7	0, 1, 2	O07	0.007
8	0, 1, 2	O14	0.014
9	0, 1, 2	O21	0.021
10	0, 1, 2	Normal	/

**Table 4:** Accuracy of model transfer experiment.

Test Number	The Source Domain–The Target Domain					
	A–B	B–A	A–C	C–A	B–C	C–B
1	97.2	97.6	96.9	96.4	98.1	98
2	96.6	96.4	97.3	97.9	97.2	97.6
3	97.1	97.7	98.5	98.1	100	99.4
4	95.4	95.2	99.1	98.8	99.3	98.9
5	96.3	96.9	98.6	98.2	96.4	96.8
6	98.6	98.4	97.8	97.5	98.4	98
7	97.6	97.1	96.6	96.3	96.5	96.6
8	98.5	98.9	95.6	95.1	97.2	97
9	98.4	98.1	97.2	97.5	95.8	96.1
10	99.1	98.8	98.3	98.5	97.9	97.5

**Table 5:** Accuracy under different noise conditions (%).

Signal-to-Noise Ratio (dB)	−3	−1	0	1	3
Accuracy (%)	85.0	90.2	92.1	95.4	96.0

**Table 6:** Specific parameters of rolling bearing.

Contents	NU205	N205
Bearing inner diameter	25 mm	25 mm
Bearing outer diameter	52 mm	52 mm
Bearing roller diameter	7 mm	7 mm
Bearing width	15 mm	15 mm
Contact angle	0 rad	0 rad
The number of the rollers	11	10
Inner-race defect (width × depth)	0.3 × 0.25 mm Early stage	
Rolling-element defect (width × depth)		0.5 × 0.15 mm Early stage
Outer-race defect (width × depth)		0.3 × 0.25 mm Early stage

**Table 7:** Rolling bearing fault information.

Tab	Speed (rpm)	Fault Type
1	600	Inner-sphere fault
2	600	Rolling-element fault
3	600	Outer-sphere fault
4	800	Inner-sphere fault
5	800	Rolling-element fault
6	800	Outer-sphere fault
7	1000	Inner-sphere fault
8	1000	Rolling-element fault
9	1000	Outer-sphere fault
10	800	Normal state

## Data Availability

The CWRU dataset is available at https://engineering.case.edu/bearingdatacenter. The JNU dataset is available at https://github.com/ClarkGableWang/JNU-Bearing-Dataset (accessed on 19 May 2024).
